# Isolation, Genotyping, and Mouse Virulence Characterization of *Toxoplasma gondii* From Free Ranging Iberian Pigs

**DOI:** 10.3389/fvets.2020.604782

**Published:** 2020-11-20

**Authors:** Mercedes Fernández-Escobar, Rafael Calero-Bernal, Javier Regidor-Cerrillo, Raquel Vallejo, Julio Benavides, Esther Collantes-Fernández, Luis Miguel Ortega-Mora

**Affiliations:** ^1^Salud Veterinaria y Zoonosis (SALUVET) Group, Animal Health Department, Faculty of Veterinary Sciences, Complutense University of Madrid, Madrid, Spain; ^2^SALUVET-innova S.L., Faculty of Veterinary Sciences, Complutense University of Madrid, Madrid, Spain; ^3^Mountain Livestock Institute, Consejo Superior de Investigaciones Científicas-Universidad de León (CSIC-ULE), León, Spain

**Keywords:** *Toxoplasma gondii*, Iberian pigs, isolates, genotypes, virulence

## Abstract

The present study aimed to isolate and perform molecular and phenotypic characterization of *Toxoplasma gondii* strains infecting Iberian pigs bred under semi-free conditions and destined for human consumption. Blood and heart tissue samples from 361 fattening pigs from 10 various herds selected in the main areas of Iberian pig production were collected at a slaughterhouse; the sera were tested for anti-*T. gondii* antibodies using a commercial indirect ELISA kit, and a mouse bioassay was carried out using heart muscle of seropositive individual representatives from each geographical location. Seventy-nine (21.9%) of the 361 animals tested positive for anti-*T. gondii* antibodies according to the serology test. Fifteen samples of myocardial tissue were subjected to bioassay and 5 isolates (TgPigSp1 to TgPigSp5) were obtained. The isolates were characterized by using 11 PCR-RFLP genetic markers; three isolates had a ToxoDB #3 genotype (3/5) and two isolates had a ToxoDB #2 genotype (2/5). The TgPigSp1 and TgPigSp4 isolates were selected for virulence in mice characterization as instances of each different RFLP-genotype found. The TgPigSp1 isolate (#2 genotype) was virulent in mice with notable cumulative mortality (87.5%) and morbidity rates (100%); the TgPigSp4 (#3) was nonvirulent and triggered mild clinical signs in 42.1% of seropositive mice. Infection dynamics and organ distribution of both isolates were analyzed; the data revealed significant differences, including substantially higher parasite load in the lung during the acute phase of infection, in mice infected with TgPigSp1 than in the case of TgPigSp4 (median parasite load 7.6 vs. 0 zoites/mg, respectively; *p* < 0.05). Furthermore, degrees of severity of detected histopathological lesions appeared to be related to higher parasite burdens. Taking into account the unexpectedly high mortality rate and parasite load associated with the clonal genotype III, which is traditionally considered nonvirulent in mice, the need for further investigation and characterization of the *T. gondii* strains circulating in any host in Europe is emphasized.

## Introduction

The eukaryotic parasite *Toxoplasma gondii* (Apicomplexa) can infect virtually all warm-blooded animals and constitutes a specific risk for food safety in the European Union (1–3); additionally, it is considered the second causal agent of foodborne illness in the USA (4). Generally, human infections are mainly acquired after ingestion of raw or undercooked meat containing viable *T. gondii* tissue cysts (5). Infections are dramatically associated with reproductive failure in pregnant women, neurological signs in immunocompromised patients, and ocular disease in otherwise healthy humans. Thus, control of *T. gondii* presence in meat destined for consumption is of major interest (2, 6). After chicken, pork meat is the most frequently consumed protein source in western countries, and Spain is the top producer of pork within the EU (7).

Black Iberian pigs (*Sus scrofa*) constitute a traditional and well-adapted pig breed whose production is linked to highly valuable meat products, especially cured ham and sausages. These animals are usually reared in extensive systems in southwestern areas of the Iberian Peninsula (covering Portugal and Spain), within a favorable ecosystem, called Dehesa, composed mostly of acorn Mediterranean forest with a high natural biodiversity, which is ideal for swine breeding (8) in sympatry with ruminant livestock and a number of other wild animals, such as Cervidae and wild boar.

Natural infections in pigs are usually asymptomatic. In Iberian pigs raised in Spain, anti-*T. gondii* antibodies have been detected with a frequency from 9.5 to 58.2% (9, 10); extensive management systems and facilities with outdoor access are associated with higher seroprevalence (11, 12); thus, parasite isolation and characterization of the circulating strains are of major interest. Currently, only a few studies reported the genotypes and virulence degrees of *T. gondii* strains infecting domestic pigs in Europe; thus, the aim of the present study was to isolate and characterize *T. gondii* strains in domestic Iberian pigs bred in the semifree systems in the traditional raising area of Dehesa located in the southwestern part of the Iberian Peninsula.

## Materials and Methods

### Ethical Statement

Animal procedures for the *T. gondii* isolation by bioassay in mice and evaluation of virulence degree (PROEX 274/16) were approved by the Animal Welfare Committee of the Community of Madrid, Spain, following proceedings described in Spanish and EU regulations (Law 32/2007, R.D. 53/2013, and Council Directive 2010/63/EU). All animals used in this study were handled in strict accordance with good clinical practices, and all efforts were made to minimize the suffering. As a humane endpoint, mice with a severe loss of body condition or nervous clinical signs were sacrificed to limit unnecessary suffering.

### Mice

Seven-week-old female Swiss/CD1 mice were obtained from a commercial supplier (Janviers Labs, Le Genest-Saint-Isle, France). The animals were free from common viral, parasite, and bacterial pathogens according to the results of the routine screening analyses performed by the manufacturer. Mice were housed with *ad libitum* access to food and water in a controlled environment with 12-h light and 12-h dark cycles, and the experimental procedures were performed at 8 weeks of age.

### Sample Collection and Serological Diagnosis for Tissue Selection

A total of 361 paired blood and myocardial tissue samples were collected from December, 2017 to June, 2018 from fattening Iberian pigs slaughtered for human consumption at an authorized slaughterhouse in Salamanca Province (western Spain) (Table 1). The blood samples were collected using BD PLUS serum tubes (Vacutainer; BD, Franklin Lakes, USA) at the bleeding step after animal stunning (Council Regulation (EC) No 1099/2009), and the apical part of the heart was sampled during the evisceration process; the samples were stored individually at 4°C until analysis. The serum was obtained after blood centrifugation and stored frozen (−20°C) until serological testing.

**Table 1:** Summarized data on geographical area of origin, number of samples collected, ELISA results and isolate obtaining from Iberian fattening pigs.

**Location of origin (province)**	**Breeding area within Spain**	**No. of serum samples analyzed**	**% of positive serum samples**	**ELISA PP**	**Isolate ID**
Navas de la Concepción (Sevilla)	South	50	14.0% (7/50)	133.3	TgPigSp1
				132.0	–
				62.1	–
Jerez de los Caballeros (Badajoz)	Southwestern	68	45.6% (31/68)	111.5	TgPigSp2
				112.0	–
				116.2	–
				110.1	–
				109.3	–
Fuente del Maestre (Badajoz)	Southwestern	34	67.6% (23/34)	111.9	TgPigSp3
				116.6	TgPigSp4
				113.8	TgPigSp5
Constantina (Sevilla)	South	20	15.0% (3/20)	55.7	–
				41.5	–
Badajoz (Badajoz)	Southwestern	14	57.1% (8/14)	–	–
San Nicolás del Puerto (Sevilla)	South	20	10.0% (2/20)	–	–
Arroyomolinos de Montánchez (Cáceres)	West	50	6.0% (3/50)	30.9	–
Monesterio (Badajoz)	Southwestern	40	0% (0/40)	–	–
Valdelosa (Salamanca)	West	14	14.3% (2/14)	30.3	–
Fuente Obejuna (Córdoba)	South	51	0% (0/51)	–	–
Total	–	361	21.9% (79/361)	–	–

*Toxoplasma gondii*-specific IgG antibody levels in swine serum samples were measured using a commercial ELISA kit (PrioCHECK® Toxoplasma Ab SR, Prionics Schlieren-Zurich, Switzerland) (cut-off at 20 for percentage of positivity -ELISA PP-). Only myocardial tissues associated with the highest ELISA PP values and representative of the widespread locations (farms of origin) (Table 1) were selected for isolation by mouse bioassay (*n* = 15).

### Bioassay in Mice and *in vitro* Cultivation

Portions of the heart muscle (50 g/each) from selected seropositive animals were subjected to acid-pepsin artificial digestion (13) prior to subcutaneous inoculation into 3 female Swiss/CD1 mice (Janvier-Labs, Le Genest-Saint-Isle, France). Mice were observed daily, and clinical signs were scored. Procedures of confirmation of mouse infection and *in vitro* isolation in cell culture were carried out as described in reference (14).

### Genetic Characterization of *T. gondii*

Genomic DNA was extracted from the cell culture-derived *T. gondii* tachyzoites of all five isolates obtained using the Maxwell® 16 mouse tail DNA purification kit (Promega, Alcobendas, Spain). Genotyping was performed by the PCR-restriction fragment length polymorphisms (RFLP) method using 11 markers as described by earlier (15, 16). Clonal type reference strains of *T. gondii* were also included in the genotyping (TgRH, type I; TgMe49, type II; and TgNED type III). RFLP-genotype numbers were assigned according to the ToxoDB database (https://toxodb.org/toxo/).

### Assays of Virulence in Mice

Two *in vivo* experiments were set for the evaluation of cumulative morbidity and mortality rates at 42 dpi (days post-inoculation) (assay A), and the tropism and burden reached by the isolates in mouse tissues during the acute and chronic stages of the infection (assay B).

#### Parasites and Inocula Preparation

The isolates TgPigSp1 and TgPigSp4 were selected as the instances of each RFLP-genotype detected in the study. Tachyzoites used for *in vivo* assays were harvested at low passages (p7 and p8, respectively) from cultures of Vero cells, when the majority of the parasites were still intracellular, and purified by filtration through a 3-μm polycarbonate filter (IpPORE®, IT4IP, Louvain-la-Neuve, Belgium) as described previously (17). The quantity and viability of tachyzoites were determined by Trypan blue exclusion followed by direct counting in a Neubauer chamber. Serial dilutions in PBS were performed to obtain the doses from 10^5^ to 1 tachyzoite(s) of each isolate per 200 μl.

#### Assay A

Each dose was intraperitoneally (IP) inoculated into five 8-week-old female Swiss/CD1 mice. Five control mice were inoculated with 200 μl of PBS. Mice were monitored twice daily for 6 weeks and clinical signs were recorded. Cumulative morbidity rate was evaluated establishing a clinical sign scoring adapted from reference (18). Cumulative mortality rate was calculated based on the ratio of casualties to the total number of infected mice (17). Serum samples from mice, which were humanely euthanized, presented sudden death, or reached the end of the experiment at 6 weeks post-inoculation, were collected and stored at −20°C until anti-*T. gondii* antibodies detection by IFAT to confirm the infection. Alternative procedures to evidence animal infection were carried out as described in reference (14).

#### Assay B

An additional group of 10 mice per isolate was IP-inoculated with 10^3^ tachyzoites; five animals were sacrificed at 7 dpi and the remaining five mice were sacrificed at 30 dpi to mimic the acute and chronic phases of the infection, respectively. Selected organs were collected during necropsies for *T. gondii* DNA detection and quantification. Briefly, mice were bled and the right cerebral hemisphere, the right eye, the right lung, half of the heart, a piece of a liver lobe, and the right kidney from each mouse were transferred immediately following euthanasia to clean 1.5 mL tubes and stored at −80°C until DNA extraction. Samples from the brain, lung, heart, liver, kidney, quadriceps femoris muscle, and tongue were fixed in 10% buffered formalin and processed for conventional histological examination. After staining with haematoxylin/eosin, lesions in the samples were subjectively categorized from 0 (no lesion) to 3 (the most severe grade within observed lesions). Serum samples were also collected and stored at −20°C until analysis.

#### Indirect Fluorescent Antibody Test (IFAT)

Detection of anti-*T. gondii* IgG antibodies in the sera was carried out by indirect fluorescent antibody test (IFAT) (19), using an anti-mouse IgG conjugated to FITC (Sigma-Aldrich) diluted 1:64 in Evans blue (Sigma-Aldrich); the cut-off was set at 1:25. Serum samples from previously known positive and negative animals were included as the controls. Tachyzoites of the Me49 strain were used as the coating antigen.

#### DNA Extraction, Detection, and Quantification

For parasites quantification, genomic DNA was extracted from 50 to 80 mg of selected tissues using a Maxwell® 16 mouse tail DNA purification kit (Promega). DNA samples were adjusted to 20 ng/μl. Screening for the *T. gondii* DNA presence was carried out by single-tube nested PCR (nPCR) amplification of the specific *ITS-1* region as described previously (20). In samples with confirmed *T. gondii* presence according to nPCR, parasites were quantified by qPCR using primer pairs for the 529-bp repeat element of *T. gondii* (21). Reactions were performed in a final volume of 25 μl using GoTaq® qPCR master mix (Promega), 10 pmol of each primer and 100 ng of DNA in an Applied Biosystems 7500 FAST real-time PCR system (Applied Biosystems, Foster City, CA, USA). Amplification was performed according to a standard protocol (10 min at 95°C, 40 cycles at 95°C for 15 s, and 60°C for 1 min). The number of *T. gondii* tachyzoites was calculated by interpolating the average Ct values on a standard curve equivalent to 10^−1^ to 10^5^ tachyzoites generated by 10-fold serial dilutions of parasite DNA in a solution of mouse genomic DNA at 20 ng/μl. Parasite load was expressed as parasite number/mg of mouse tissue. Standard curves for *T. gondii* had a consistent average slope close to −3.3 with a *R*^2^ > 0.98.

In addition, due to the unexpected mortality rates observed, we conducted nested PCR for *CS3* locus amplification (16) over *Toxoplasma*-positive DNA samples from lung tissues of mice infected by TgPigSp1 (*n* = 3) and TgPigSp4 (*n* = 4) isolates (sacrificed at 7 or 30 dpi, respectively) for inocula genotype confirmation. PCR products were subjected to Sanger sequencing and obtained sequences were analyzed as described previously (14). *CS3* sequences alignment to previously obtained TgPigSp1 (MW132600), TgPigSp4 (MW132601) and other reference strains (TgRH, MW151245; TgMe49, MW151246; TgNED, MW151247) sequences was carried out using the Clustal Omega software (https://www.ebi.ac.uk/Tools/msa/clustalo/).

### Statistical Analyses

Variations in parasite burden in the target tissues between the inoculated mice groups were analyzed by pairwise comparisons using the Mann-Whitney U test. Lethal outcome in mice was analyzed by the Kaplan–Meier survival method, and the Mantel-Cox log-rank test was used to compare the resulting survival curves. The significance for these analyses was established at *p* < 0.05. GraphPad Prism 6 v.6.01 (San Diego, CA, USA) software was used to perform all statistical analyses and graphical illustrations.

## Results

### Serological Screening in Iberian Pigs and Parasite Isolation

*Toxoplasma gondii*-specific IgG antibodies were detected in 21.9% (79/361) of serum samples collected from pigs raised in 10 various locations (Table 1); 15 myocardial tissues of representative animals of various origins with the highest ELISA PP values were subjected to bioassay; subsequently, five isolates (TgPigSp1 to TgPigSp5) were obtained (Table 2). The bioassay success rate was 33.3% (5/15). The isolation rate appeared to increase at higher ELISA PP values; parasites were isolated from 0 of 6 pigs with PP values <110 and from 5 of 9 pigs with PP values > 110.

**Table 2:** PCR-RFLP genotyping of *Toxoplasma gondii* isolates from Iberian pigs.

				**PCR-RFLP alleles**^*******a*******^													
**Isolate ID**	**Sample, host (location)**	**ELISA PP**	**Mice bioassay (no. infected/no. inoculated)**	**SAG1**	**3^**′**^-SAG2**	**5^**′**^-SAG2**	**Alt. SAG2**	**SAG3**	**BTUB**	**GRA6**	**c22-8**	**C29-2**	**L358**	**PK1**	**Apico**	**CS3**	**RFLP-ToxoDB genotype #**
RH	CNS, human (EEUU)	–	–	I	I/III	I/II	I	I	I	I	I	I	I	I	I	–	10
Me49	Muscle, sheep (EEUU)	–	–	II/III	II	I/II	II	II	II	II	II	II	II	II	II	–	1
NED	Placental tissues, human (France)	–	–	II/III	I/III	III	III	III	III	III	III	III	III	III	III	–	2
TgPigSp1	Myocardium, pig (Sevilla, Spain)	133.3	(1/3)	II/III	I/III	III	III	III	III	III	III	III	III	III	III	III	2
TgPigSp2	Myocardium, pig (Badajoz, Spain)	111.5	(2/3)	II/III	I/III	III	III	III	III	III	III	III	III	III	III	III	2
TgPigSp3	Myocardium, pig (Badajoz, Spain)	111.9	(1/3)	II/III	II	I/II	II	II	II	II	II	II	II	II	I	II	3
TgPigSp4	Myocardium, pig (Badajoz, Spain)	116.6	(3/3)	II/III	II	I/II	II	II	II	II	II	II	II	II	I	II	3
TgPigSp5	Myocardium, pig (Badajoz, Spain)	113.8	(3/3)	II/III	II	I/II	II	II	II	II	II	II	II	II	I	II	3

a *I, II or III refers to the archetypal alleles from a Type I, II or III, for each molecular marker (15)*.

### Genetic Characterization

Cell culture-derived tachyzoites from all five isolates were successfully characterized as having two different genotypes: ToxoDB #3 (3/5 isolates) and ToxoDB #2 (2/5). The *CS3* marker was previously reported to have a high predictive value on virulence in mice (16), and was present in type II alleles in all isolates with the ToxoDB #3 genotype; type III alleles were detected in all isolates with the ToxoDB #2 genotype (Table 2).

### Pathogenicity Characterization

**Figure 1:**
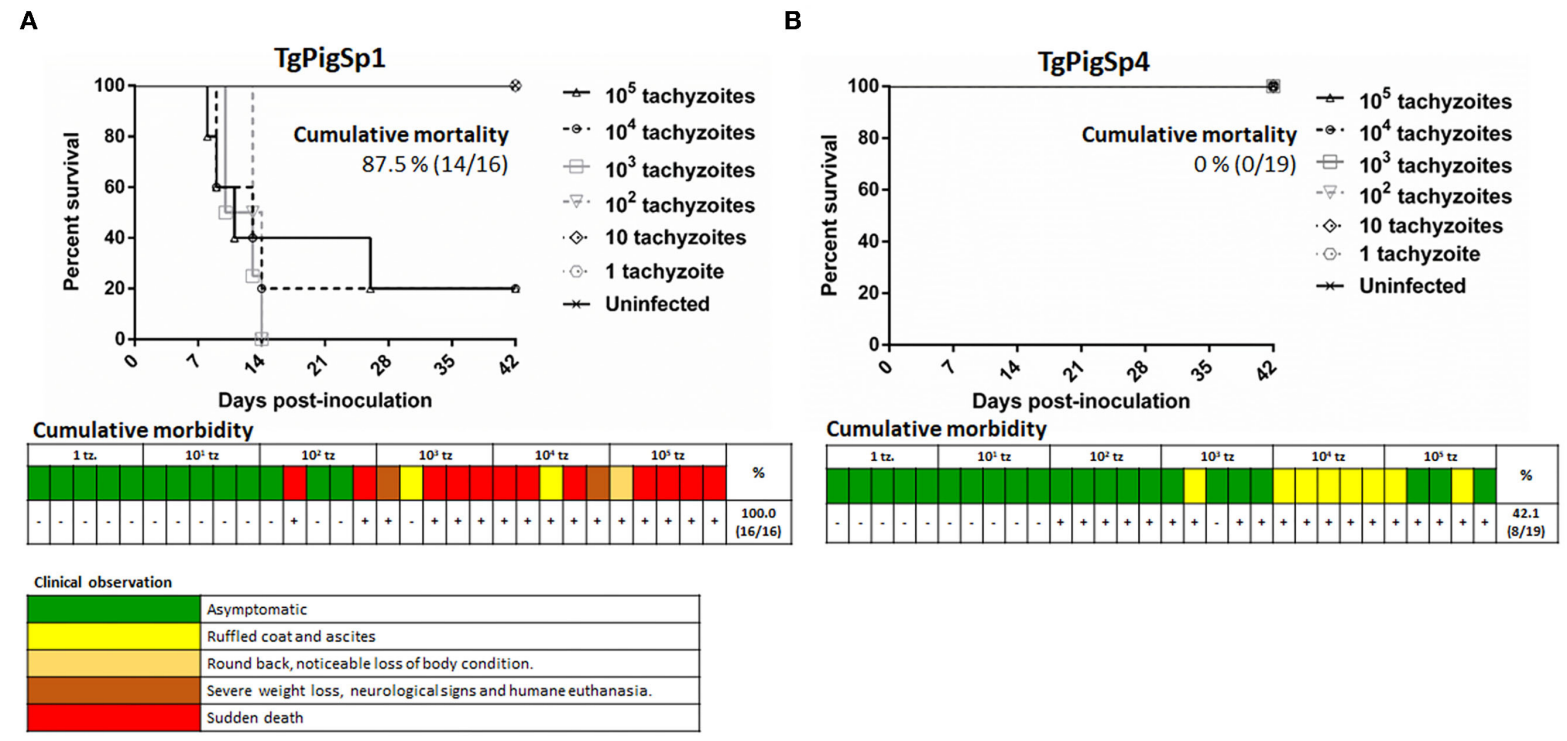
Cumulative mortality and morbidity parameters in the evaluation of virulence degree in two *Toxoplasma gondii* isolates from Iberian pigs. Survival curves and gradation of clinical signs observed in mice infected/inoculated with TgPigSp1 **(A)** and TgPigSp4 **(B)** isolates are shown. Colored boxes represent the most severe clinical sign observed in each individual mouse challenged with each dose according to clinical signs scoring included down below. +: mice with a confirmed *Toxoplasma*-infection by IFAT or tissue imprints of brain or lung. -: mice without a confirmed *Toxoplasma*-infection. Cumulative mortality rates are indicated.

In assay A, the cumulative mortality rate was 87.5% for TgPigSp1 and 0% for the TgPigSp4 isolates, as shown in the survival curves (Figure 1A). Clear and statistically significant differences were observed between the survival curves of the two isolates at the doses starting from 10^2^ tachyzoites/mouse (*p* < 0.01). Regarding morbidity rate, maximum clinical signs score reached by each animal is shown in Figure 1. In detail, the TgPigSp1 isolate caused clinical signs in 100% (16/16) of infected mice at a very acute phase of the infection at the doses starting from 10^2^, rapidly inducing rounded back and noticeable loss of body conditions in the majority of mice (93.8%, 15/16); severe weight loss and development of neurological signs (with consequential humane euthanasia) (12.5%, 2/16) were observed with frequent cases of sudden death (75%, 12/16) (Figure 1A). All sudden deaths or humane euthanasia episodes occurred before 15 dpi, except an animal that died at 26 dpi. On the other hand, the TgPigSp4 isolate triggered clinical signs in 42.1% (8/19) of infected mice, specifically including ruffled coat and ascites in all cases during a very acute phase of the infection; however, the clinical signs receded completely at 13 dpi (Figure 1B).

**Figure 2:**
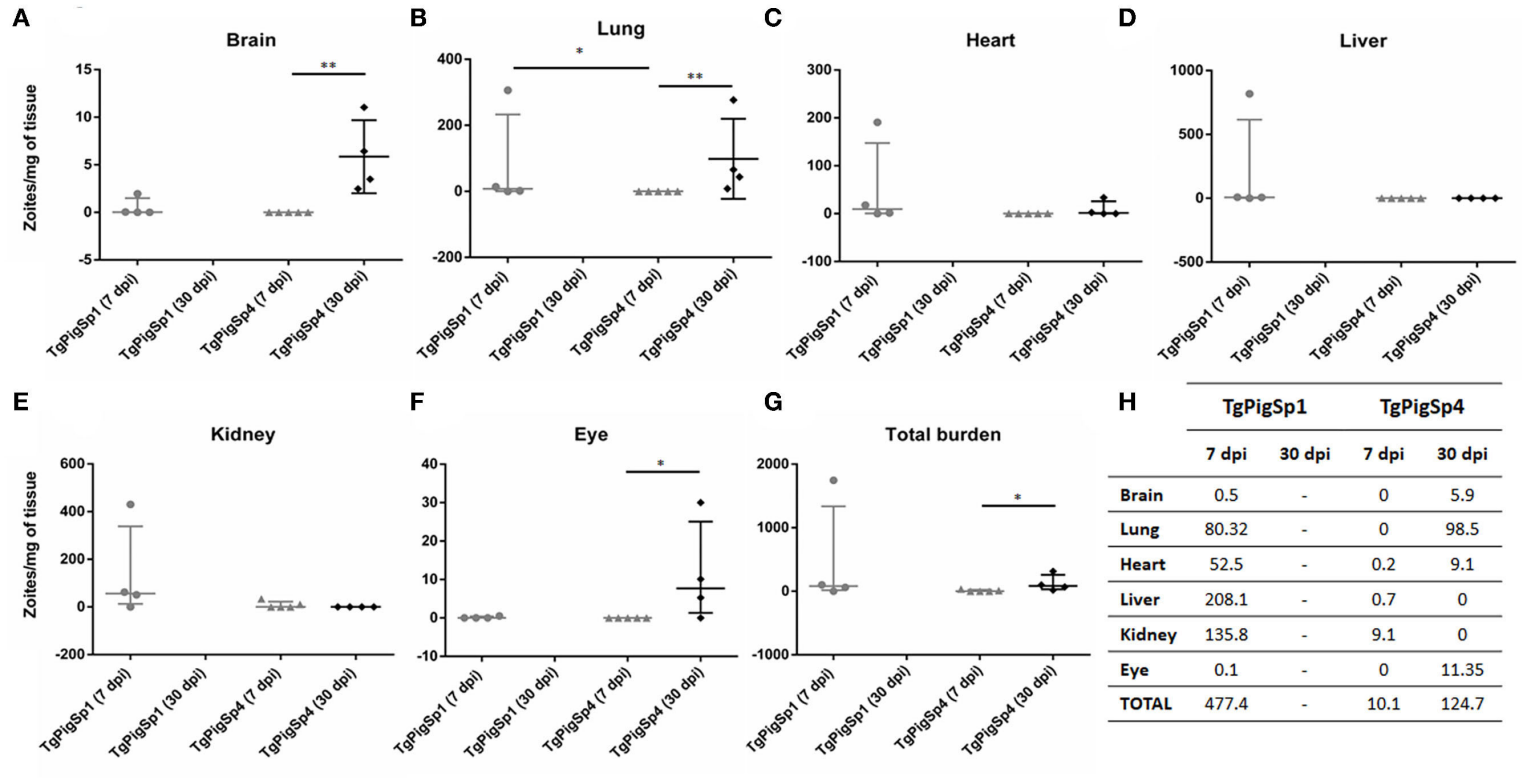
Parasite load (zoites/mg of tissue) found in six different murine organs **(A–F)** and total parasite burden per mouse **(G)** at 7- or 30-days post inoculation with 1,000 *Toxoplasma gondii* tachyzoites of TgPigSp1 and TgPigSp4 isolates. Median and interquartile values are represented. U-Mann-Whitney test; *p < 0.05; **p < 0.01. **(H)** Parasite burden (zoites/mg of tissue) mean values reached in each organ studied at 7 and 30 dpi by TgPigSp1 and TgPigSp4 isolates were tabulated.

Parasite burden reached by both isolates in the different organs studied at 7 and 30 dpi (assay B) is shown in Figure 2. All mice from the negative control group were seronegative and no *Toxoplasma*-DNA was detected by PCR in their tissue samples. *CS3* sequences amplified from *Toxoplasma*-positive DNA samples from lung tissues of infected mice were aligned to previously obtained sequences from TgPigSp1 (MW132600), TgPigSp4 (MW132601) and other reference strains (TgRH, MW151245; TgMe49, MW151246; TgNED, MW151247). 100% identity was found between *CS3* sequences got from TgPigSp1-infected tissues, that from the original isolate and TgNED sequence; likewise, all *CS3* sequences from TgPigSp4-infected tissues were identical to TgPigSp4 isolate original one and to TgMe49 sequence, confirming the correct genotype of each inoculum. Only 2 out of 5 mice infected by TgPigSp1 isolate and scheduled for sacrifice at 30 dpi survived until that point; however, they were not seroconverted and did not have parasite DNA in their tissues. This fact revealed a low success rate of infection achieved, probably due to variations inherent to proceedings (e.g., slight differences in inocula or animal individual susceptibility), and supported the high mortality rate exposed previously in the case of TgPigSp1, which made not possible to compare the parasite burdens reached by each isolate at 30 dpi. At 7 dpi, all tested organs had higher average parasite burden in mice infected with TgPigSp1 than that in mice infected with TgPigSp4 isolate (Figure 2H); nevertheless, due to high variance of the data, differences were significant only between the parasite loads in the lung tissues, where TgPigSp1 infection had a median parasite load of 7.6 zoites/mg, whereas no *T. gondii* DNA was detected in mice infected with TgPigSp4 (*p* < 0.05) (Figure 2B). In the eye, at the acute stage, *T. gondii*-DNA was detected only in a mouse infected with TgPigSp1 (0.5 zoites/mg). Usually, *T. gondii* eye infection is not bilateral; detection by nPCR was performed only in the right eye of each animal thus limiting the frequency of detection. Statistically significant differences were detected in mice infected with TgPigSp4 at 7 vs. 30 dpi in terms of parasite burden in the brain (median 0 vs. 5 zoites/mg; *p* < 0.01), lung (median 0 vs. 54.7 zoites/mg; *p* < 0.01) and ocular tissues (median 0 vs. 7.7 zoites/mg; *p* < 0.05); no significant differences were detected in the case of heart, liver, and kidney tissues. Overall, total parasite burden reached in mice infected with TgPigSp4 was significantly increased from 7 to 30 dpi (median 0.7 vs. 83.3 zoites/mg; *p* < 0.05).

**Figure 3:**
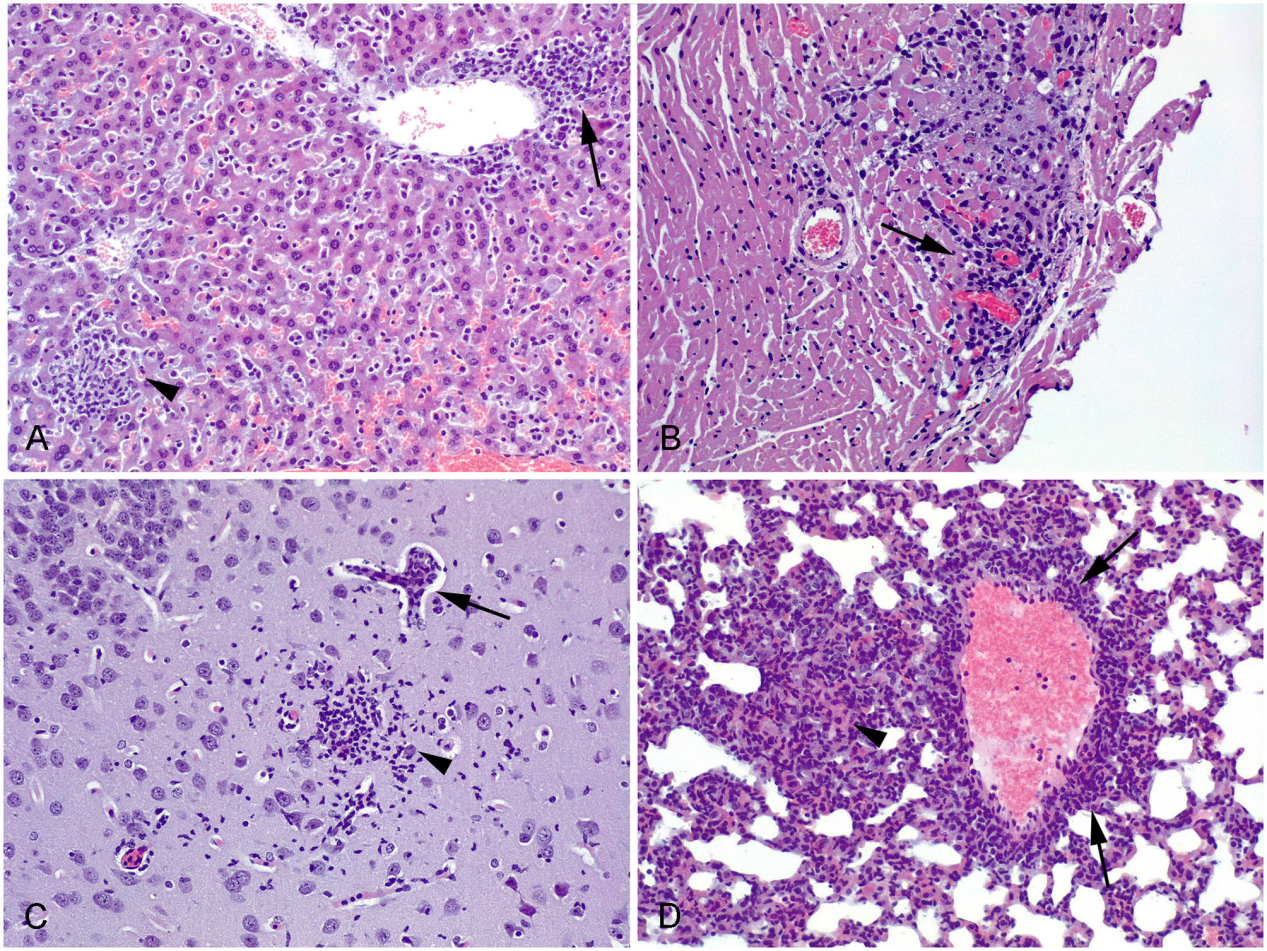
Histological lesions observed in *Toxoplasma gondii* infected mice. HE. 20 x. **(A)** Liver. Mouse infected with TgPigSp4, 7 dpi. Aggregation of mononuclear inflammatory cells at the hepatic parenchyma (arrowhead) and in relation with a central vein (arrow). **(B)** Heart. Mouse infected with TgPigSp1, 14 dpi. Foci of non-purulent myocarditis (arrow). **(C)** Brain. Mouse infected with TgPigSp4, 30 dpi. There is a glial focus at the neuropile (arrowhead) close to a small vessel showing non-purulent vasculitis (arrow). **(D)** Lung. Mouse infected with TgPigSp4, 30 dpi. Perivascular infiltration of nonpurulent inflammatory cells (arrows). A focal thickening of the alveolar wall, caused by the infiltration of inflammatory cells, could also be observed (arrowhead).

In all histologically evaluated organs, lesions were mainly observed in the brain, liver, and lung, where multifocal aggregates of mononuclear inflammatory cells were detected (Figure 3). No lesions were found in the kidney, quadriceps femoris muscle or tongue tissue. Similar to the parasite load assessment, due to the high mortality rate caused by TgPigSp1, it was not possible to compare the tissue lesion patterns triggered by each isolate at 30 dpi. Organs of three seropositive mice scheduled to be sacrificed at 30 dpi that experienced early sudden dead or were humanely culled, were included for histopathological evaluation to provide further evidence of TgPigSp1 infection course; however, the results were not useful for comparison. In the liver of mice infected with TgPigSp1 lesions were subjectively more severe at an early phase of infection (taking into account the tissue samples from mice sacrificed at 7 dpi and samples from mice who died early at 11–14 dpi (four mice in total) than of those infected with TgPigSp4 (only one animal had grade 3 lesions at 7 dpi). Moreover, the only mouse with heart lesions (i.e., multifocal nonpurulent myocarditis, Figure 3) was infected with TgPigSp1 and died at 14 dpi. On the other hand, the lesions in the brain were distinguished by glial foci and perivascular infiltration of inflammatory cells, and were detected in two mice infected with TgPigSp4; this phenomenon can be explained by development of chronic infection induced by TgPigSp4 isolate unlike acute infection caused by TgPigSp1. Regarding the lung, only 2 mice (one infected by each isolate) had histopathological lesions at 7 dpi; the lesions were more notable in the case of the TgPigSp1 infection. Furthermore, a TgPigSp1-infected animal with evidence of myocarditis (died at 14 dpi) also presented inflammatory lesions in the lung. Finally, 4 out of 5 mice inoculated with TgPigSp4 isolate had multifocal aggregates of mononuclear cells in the lung at 30 dpi.

## Discussion

The present study aimed to isolate, genotype, and evaluate the virulence degree in a normalized mouse model of the *T. gondii* strains infecting Iberian pigs. Seroprevalence values observed in the present study are in agreement with the previous data (9.5–58.2%) reported in the same host and breeding systems in Spain (9, 10, 22). Age and management systems are important factors related to an increase in the seroprevalence levels (11), which makes free-ranging fattening Iberian pigs a perfect model for *T. gondii* isolation and for evaluation of consumer risk when trends in organic pig production are concerned with natural breeding and welfare. The observed higher success of parasite isolation from the animals with higher ELISA titers should be considered in the future surveys.

Genetic characterization based on PCR-RFLP identified 2 genotypes, ToxoDB #3 (type II PRU-variant) and #2 (clonal type III), predominantly identified in the European domestic livestock (reviewed in (14)), wildlife (23) and humans (24–26). In Europe, the available data on genotyping of *T. gondii* in infected pigs is scarce; recently, an study (27) summarized the major facts, including the presence of all three clonal lineage alleles with an apparent predominance of type II; however, it should be noted that in most of studies, only one or a few molecular markers was used thus limiting the resolution of the results. A unique study using the same 11 molecular markers reported unexpected combinations of I, II and III alleles in 11 tissue samples from organic pigs in Italy (28). Notable frequency of type III alleles has been also described in Portuguese and Italian studies (29, 30); nonetheless, the studies were based only on *SAG2* PCR-RFLP and five microsatellite loci (*TUB2, TgM-A, W35, B17*, and *B18*) or on *B1*-PCR-HRM (high resolution melting) genotyping assays, respectively. Similarly, a recent Serbian study reported that 22.2% (2/9) of detected strains correspond to the genotype III according to PCR-RFLP of *GRA6, alt. SAG2, PK-1, BTUB, C22-8, CS3* and *Apico* markers (31); nevertheless, one of the isolates had a recombinant type II allele in the *C22-8* marker. A Polish survey detected high prevalence of type III alleles in *T. gondii* DNA extracted from retail raw pork meat products (32). Similar results were obtained in the myocardial tissues of wild boars ranging in the southwestern locations in Spain (33) close to the sampling areas covered in the present study. Surprisingly, a high frequency of type I alleles was described in European literature (27, 34, 35) although type I were not detected in the present study except *Apico* marker identified within the Pruginaud (PRU) variant of the clonal genotype II. Remarkably, ToxoDB #3 genotype (type II PRU-variant) had not been described previously in studies using similar methodologies in infected European pigs (28, 31, 36). Different typing methodologies and sampling efforts used in the available references of the literature complicate the conclusions; however, it appears that overlapping strains from the three clonal genotypes are infecting European pig livestock, and recombination events within feline population are possible (28, 35, 36). This fact may be favored by the production systems involving pigs raised in semi-free ranging conditions that are exposed to potential sources of *T. gondii*, e.g., oocyst-contaminated environment or animal carcasses (37).

Currently, only two studies (29, 31) reported isolation of *T. gondii* from domestic swine in Europe; a third study (38) reported the seroconversion and DNA detection in the brain of mice inoculated with the homogenates of meat from 16 seropositive pigs reared in the indoor systems in the Aragon region of Spain. In wild swine, viable *T. gondii* were isolated from 21 wild boar heart tissue samples in France (23); all samples were identified as genotype II by PCR-RFLP (*SAG1, SAG2*, and *GRA7*) and microsatellite loci analysis (*TUB2, TgM-A, W35, B17, B18, and M33*). No additional data were reported thus increasing the interest in the present investigation along with the fact that no previous assays aimed to determine the virulence of European isolates from pigs.

The *CS3* locus has been described previously as a highly predictive marker of mortality in mice inoculated with *T. gondii* isolates (16). Our *CS3* typing results disagree with previous Brazilian and Chinese studies reporting high mortality rates associated with the type I or II alleles of the *CS3* gene and low or null rates associated with the type III alleles (16, 39, 40). However, opposite results have been reported in studies from Brazil (41, 42) and Spain (14). Clearly, additional investigations are required to determine the role of this locus and its interaction with related well-known virulence factors, such as ROP5 and ROP18 (43), in *Toxoplasma* virulence in mice and other hosts.

In this study, an apparent correlation between the genotype and mortality and morbidity rates in mice is described; nonetheless, further studies are needed. *Toxoplasma gondii* clonal lineages I, II and III have been traditionally classified according to their virulence in outbred laboratory mice into highly virulent (type I), intermediate virulent (type II) and nonvirulent (type III) (44–46). Nevertheless, current population structure of *T. gondii* is not limited to the three clonal lineages and is considerably more complex with at least 16 haplogroups known worldwide (47, 48); moreover, pre-established virulence classification is apparently also disputed. In the present study, an isolate was classified as clonal type III according to 11 RFLP-markers (TgPigSp1) and therefore was expected to be nonvirulent; however, the isolate had 87.5% cumulative mortality in the standardized mouse model, whereas a type II isolate (genotype #3, TgPigSp4) was completely nonvirulent while it has been described as moderately virulent previously. In the case of the TgPigSp1 isolate, already starting from the dose of 10^2^ tachyzoites/mouse, the mortality rates were 80–100%. High mortality rate detected in the present study is in agreement with a previous report from Japan, where oral doses of 10^2^-cyst of a type III cat isolate were found to cause 100% mortality in mice; nevertheless, no clinical signs of infection were seen when micro minipigs were infected in a similar manner (49). Although a relative correspondence between the virulence of *T. gondii* strains in mice and their virulence in humans is traditionally assumed (50), there are many studies that point out different behavior of the parasite in different hosts (49, 51, 52). Thus, the mouse virulence model should be interpreted as a relative and not an absolute characterization method; therefore, the mortality values described here will be valid for a mouse model but not for assessing the virulence degree of the TgPigSp1 and TgPigSp4 isolates in a swine model. Increasing evidence of a host-dependent virulence and a broken linkage with genotype, emphasize the need to investigate still unknown *T. gondii* strains virulence factors.

The parasite load is associated with the histological lesions detected in the tested mouse tissues, indicating a higher degree of virulence of the TgPigSp1 isolate compared to that of the TgPigSp4 isolate thus supporting the results of the cumulative mortality and morbidity rates. A high incidence of the lesions in the tissues of mice infected by TgPigSp4 at 30 dpi, especially in the brain and lung, corresponds to the development of a chronic infection similar to mice that were infected with low virulence type II isolates (53); however, high cumulative mortality of TgPigSp1 does not permit any additional comparison.

In addition to the cumulative morbidity and mortality rates, the virulence degree can be evaluated by nonlethal infection parameters; a pioneering study identified the intra-genotype variations in the weight gain and both anti-*T. gondii* IgG antibodies and haptoglobin levels in the serum of mice infected with a panel of type II isolates (54). Interestingly, variations in these parameters were observed in strains of the same genotype isolated from different hosts. Recent evaluation of the virulence degree in Caribbean, Brazilian and European *T. gondii* strains of six different genotypes showed a wide variability in the mortality rate and in parasite burden in the mouse tissues at 8 dpi (55). Additional studies aiming to combine genotypic and phenotypic characterization of the European isolates will be of major interest to determine the possible presence of hidden intragenotype variations within the clonal *T. gondii* strains, which were formerly classified as moderately virulent (type II) and nonvirulent (type III).

It should be noted that the isolates included in the present experiments on phenotypical characterization have been obtained recently (low number of cell culture passages), fact that enables to conserve their *in vivo* biological behavior avoiding the adaptation to the cell culture conditions. This feature is important since various factors, such as life stage of the parasite or the number of passages in mice or in cell culture, have been repeatedly demonstrated in the literature to notably influence the evaluation of the virulence parameters (17, 49, 52).

The present study provides new information on the *T. gondii* strains circulating in swine in Europe and opens interesting avenues toward the epidemiological importance of trending organic farming and semi-free systems with regard to food safety, especially when domestic pigs are raised in sympatry with wildlife; in this sense, studies on the circulation of *T. gondii* from a One Health approach are of major interest. Considerable sampling and isolation efforts along with genotyping improvements were made to change the paradigm of the genetic structure of *T. gondii* population (56); similarly, implementation of virulence/phenotypical characterization of a large number of *T. gondii* strains by accurate models, including mortality and evaluation of nonlethal infection parameters, may let us to solve the raised controversy.

## Data Availability Statement

The raw data supporting the conclusions of this article will be made available by the authors, without undue reservation.

## Ethics Statement

The animal study was reviewed and approved by Animal Welfare Committee of the Community of Madrid (PROEX 274/16).

## Author Contributions

MF-E, RC-B, EC-F, and LO-M conceived and designed the laboratory tests. MF-E, RC-B, JR-C, RV, and JB performed experiments. MF-E, RC-B, RV, JR-C, and EC-F analyzed the data. LO-M, EC-F, and JB contributed reagents, materials, analysis tools. MF-E, RC-B, JR-C, JB, EC-F, and LO-M drafted the manuscript. All authors contributed to the article and approved the submitted version.

## Conflict of Interest

The authors declare that the research was conducted in the absence of any commercial or financial relationships that could be construed as a potential conflict of interest.
